# Heparin induced bullous hemorrhagic dermatosis at a site distant from the injection. A report of five cases*

**DOI:** 10.1590/abd1806-4841.20165418

**Published:** 2016

**Authors:** Vanessa Gargallo, Fatima Tous Romero, José Luis Rodríguez-Peralto, Carlos Zarco

**Affiliations:** 1 12 de Octubre University Hospital – Madrid, Spain

Heparins both unfractionated and low-molecular-weight are associated with some cutaneous complications including hematomas, ecchymosis, erythematous plaques, nodules, skin necrosis, contact dermatitis and urticaria, all occurring more commonly at local subcutaneous injection sites.^1,2^ First reported at 2006 by Perrinaud *et al,* bullous hemorrhagic dermatosis is a rare cutaneous reaction to heparin in which hemorrhagic intraepidermal bullae appear in areas distant from the heparin injection sites and of which there are less than 20 cases described in the literature. ^1,2^

**Figure 1:**
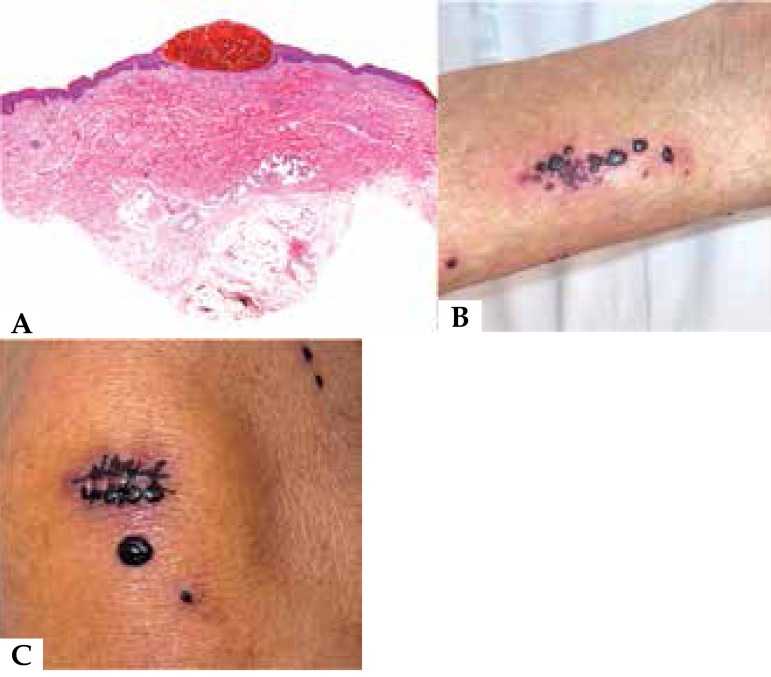
Bullous hemorrhagic dermatosis at sites distant from subcutaneous injections of heparin. **A:** Histopathologic findings: subepidermal blister filled with red blood cells. Case 1. **B:** Linear arrangement of the lesions. Case 4. **C**: Koebner phenomenon with development of new lesions on the area in which the stiches were given. Case 3

We present 5 cases of heparin induced bullous hemorrhagic dermatosis at a site distant from the injection. The characteristics of each patient are detailed in table 1. All the patients were male with mean age of 74 years, in treatment with enoxaparin at different doses. Patients 3 and 4 were also taking antiplatelet drugs. The onset of bullae was 8-20 days after the beginning of the heparin therapy and the lesions were asymptomatic in all cases. Biopsy was performed in the 5 cases, showing intraepidermic blister filled with red blood cells, without any signs of vasculitis or vessels thrombosis, and heparin-induced bullous hemorrhagic dermatoses was diagnosed (Figure 1A). Laboratory tests' results, blood count and coagulation studies were normal. Patients 2, 3, and 4 had pruritic conditions previous to the onset of lesions; therefore they scratched their skin. We observed that these patients presented more lesions and that they were more disseminated than in those patients without pruritus. What is more relevant, in patients 2, 3 and 4 some of the lesions had a linear, Koebner-like, arrangement (Figure 1B). Strikingly, patient 3 developed new lesions on the stitches at the site of biopsy (Figure 1C). Patient 5 had no pruritic condition, but the appearance of the lesions was clearly associated to an occasional scratch on the area. In three of our five cases we maintained the treatment; two of them self-resolved without discontinuation but treatment was changed in patient 2 because new lesions kept appearing, but it also had a complete resolution within few weeks. The reaction to heparin seemed to be retarded as proved by the late onset of the bullae, ranging from 8 to 20 days after the beginning of the heparin therapy. This data is also consistent with the reports previously published.^1,3^

**Table 1:** Bullous hemorrhagic dermatosis at sites distant from subcutaneous injections of heparin. Clinical features

Patient number	Sex	Age	Relevant comorbidities	Previous use of heparin	Diagnosis for heparin use	Other anticoagulants	Heparin type and doses	Latency	Number of bullae	Pruritus/other skin diseases	Linear lesions or Koebner phenomenon	Lesion location	Evolution
1	Male	90	Aortic stenosis	Yes	Aortic valve replacement	No	Enoxaparin 80mg/12h	8 days	< 5	No	No	Ankle and wrist	2 weeks; Heparin maintained
2	Male	65	Cryptogenic organizing pneumonia	No	Atrial fibrillation	No	Enoxaparin 60mg/12h	9 days	> 30	Yes. Renal insufficiency causing pruritus	Yes	Lower and upper extremities	2 months. After 1 month and a half change treatment to tinzaparin. Res-olution 2 weeks after.
3	Male	64	Ischemic cardiomyopathy	Yes	Study previous to heart transplantation	Aspirin 100mg/d	Enoxaparin 60mg/12h	7 days	> 30	Yes. Xeroderma	Yes	Lower and upper extremities	3 weeks; Heparin maintained
4	Male	89	Cardiac decompensation	No	Atrial fibrillation	Aspirin 300mg/d	Enoxaparin 40mg/12h	10 days	> 100	Yes. Chronic urticaria	Yes	Lower and upper extremities, scalp and upper part of the back	3 weeks; Heparin suspended
5	Male	74	Systemic amyloidosis	Yes	Atrial fibrillation	No	Enoxaparin 40mg/12h	20 days	< 5	No	No	Hand and leg	2 weeks; Heparin suspended

The pathogenesis of this condition is unclear. Since some patients were receiving high doses of heparin it has been proposed a dose-related reaction.^3^ In our series only one patient received very high doses of heparin, and two patients received low dose. Other authors also agree with this observation,^4^ being unlikely an overdose phenomenon. A synergic mechanism has also been proposed for patients treated with one or more anticoagulants or antiplatelet drugs, however, cases also occurred without anticoagulants and with normal coagulation studies.^1,3,4^ Only two of our patients were taking anticoagulants and coagulation studies were normal in all the cases, therefore we doubt the contribution of theses factors in the development of the bullae. Hypersensitivity reaction to heparin injection has been suggested ^4^ but the absence of eosinophils on histology does not support this theory. Previous reports also show lesions arranged in groups on small skin areas ^5^ or showing a linear, Koebner-like, arrangement.^1,4^ This consistent with the relevance of an external trauma causing or increasing the number of lesions. More recent reports indicate that in most cases discontinuation of the treatment is not necessary.^2,4^ In the cases in which we decided to maintain treatment the lesions eventually disappeared. However, in one case the lesions where persistent for a month and a half hence treatment was changed with complete resolution afterwards. Therefore if the appearance of new lesions continues for longer than three weeks it should be advisable to change the anticoagulant therapy. Our observations prove an increased number of lesions after trauma. This might not be the only cause but it is for sure an important factor in the development of the bullae, with a significant increase in the number of lesions in patients suffering from pruritic conditions. In fact, disseminated lesions emerged only in these patients. Since Koebner phenomenon occurs in this disease dermatologist should be aware that there can be disseminated and more persistent lesions in patients with pruritic conditions. An individualized approach, taking into account the extension, time of evolution and the importance of anticoagulation in the context of each patient helps to decide whether to suspend, maintain or change therapy.
